# Synergistic effects of combining proteasome inhibitors with chemotherapeutic drugs in lung cancer cells

**DOI:** 10.1186/s13104-017-2842-z

**Published:** 2017-11-02

**Authors:** Linda Sooman, Joachim Gullbo, Michael Bergqvist, Stefan Bergström, Johan Lennartsson, Simon Ekman

**Affiliations:** 1 Department of Immunology, Genetics and Pathology (former Radiation, Oncology and Radiation Science), Section of Oncology, Rudbeck Laboratory, Uppsala University, Dag Hammarskjölds väg 20, 751 85 Uppsala, Sweden; 2 Department of Medical Sciences, Division of Clinical Pharmacology, Uppsala University Hospital, 751 85 Uppsala, Sweden; 3 Center for Research & Development, Uppsala University/County Council of Gävleborg, Gävle Hospital, 801 87 Gävle, Sweden; 4 Department of Oncology, Gävle Hospital, 801 87 Gävle, Sweden; 5 Department of Radiation Sciences & Oncology, Umeå University Hospital, 901 87 Umeå, Sweden; 6 Department of Pharmaceutical Biosciences, Uppsala University, 751 24 Uppsala, Sweden; 7 Department of Oncology-Pathology, Karolinska Institutet, 171 76 Stockholm, Sweden

**Keywords:** Lung cancer, Combination chemotherapy, Proteasome inhibitors

## Abstract

**Background:**

The prognosis for patients with disseminated lung cancer is poor and current treatments have limited survival benefit as resistance often occurs, and is often associated with significant toxicity. A possible strategy to improve treatment and evade chemoresistance may be to find new combinations of drugs. The aim of this study was to analyze the potential of combining proteasome inhibitors (PIs) with chemotherapeutic drugs used in the routine treatment for lung cancer patients.

**Results:**

The median-effect method was applied to the Fluorometric Microculture Cytotoxicity Assay (FMCA) to evaluate effects of combining two different PIs (bortezomib and b-AP15) with clinically used chemotherapeutic drugs representing different mechanisms of action (cisplatin, gefitinib, gemcitabine and vinorelbine) in two lung cancer cell lines (one sensitive and one resistant). Proteasome inhibition in combination with cisplatin, gemcitabine or vinorelbine had synergistic effects in at least one of the tested cell lines. Furthermore, the effect of gefitinib appeared strongly potentiated by the PI in the least resistant lung cancer cell line, although the level of synergy could not be determined with the median-effect method.

**Conclusions:**

Combining PIs with cisplatin, gefitinib, gemcitabine or vinorelbine show potential as new combination chemotherapy for the treatment of lung cancer.

**Electronic supplementary material:**

The online version of this article (10.1186/s13104-017-2842-z) contains supplementary material, which is available to authorized users.

## Background

Lung cancer (LC) is causing most cancer-related deaths in the world and is responsible for 1.2 million new cases per year [1]. Although heterogenous, LC usually presents an aggressive, fast growing cancer which commonly metastasizes. It is a potentially curable disease when discovered at an early stage, but due to commonly late diagnosis at an advanced disease stage the 5-year survival rate is among the lowest of all cancers at 10–15% [2]. It can be categorized into two main histological subtypes; non-small cell lung cancer (NSCLC) and small cell lung cancer (SCLC). The most common sub-type is NSCLC, which constitutes at least 80% of all LCs [2]. Patients with advanced disease are generally treated with chemotherapy but the prognosis is dismal with a median survival time of only around 12–14 months and with a 5-year survival of less than 10% [3]. Current standard chemotherapy for advanced LC in the first-line includes a platinum-based doublet chemotherapy [4, 5]. Cisplatin is the preferred platinum compound, if not contraindicated, and is combined with a third-generation chemotherapeutic, including vinorelbine, gemcitabine or a taxane for NSCLC and a topoisomerase inhibitor for SCLC [5]. The discovery of oncogenic driver mutations in subgroups of NSCLC has opened up for new treatment strategies with targeted therapy. Sensitizing mutations in the kinase domain of the epidermal growth factor receptor (EGFR) leads to sensitivity to tyrosine kinase inhibitors, of which gefitinib, erlotinib and afatinib are in clinical use and are the preferred treatment of choice for EGFR mutated patients [6–8].

The ubiquitin–proteasome system is responsible for degradation of proteins by tagging them with ubiquitin which leads to recognition by the proteasome complex and degradation of the proteins into small peptides. The ubiquitin–proteasome system is important for maintenance of cell homeostasis which is critical for normal function and survival of all cells. Dysregulation of protein degradation has been shown to play an important role in the growth and survival of tumors [9] and proteasome inhibitors (PIs) have significant preclinical and clinical activity in several cancers, especially in combination with other chemotherapeutic drugs [10]. Bortezomib, a small-molecule 20S PI [11], was the first PI approved for clinical use in the treatment of multiple myeloma [12]. Bortezomib as a single agent has shown minimal activity in SCLC, but promising preclinical activity has been observed in combination with agents commonly used for LC patients [13]. From multiple myeloma treatments it is known that bortezomib treatment is associated with problematic side effects that may limit its usefulness in drug combinations (e.g. neurotoxicity), and that mutations in the 20S subunit may result in drug resistance [14]. Therefore, an alternative promising approach is to inhibit the regulatory activity of the 19S subunit. b-AP15 was recently identified as an inhibitor of 19S proteasome deubiquitinase activity [15] and has anti-cancerous effects in vitro and in animal models [16].

There is still a lack of knowledge of the optimal combinations of PIs and chemotherapeutics, their synergistic effects and potential use in resistance to chemotherapy in LC. The present work aims to address these issues with two PIs targeting the 19S or the 20S proteasome in combination with commonly used chemotherapeutics in LC representing different mechanisms of action.

## Methods

### Cell culture

The NSCLC squamous cell carcinoma cell lines U-1752 and NCI-H157, the NSCLC large cell carcinoma cell line U-1810, the NSCLC adenocarcinoma cell line NCI-H23 and the SCLC cell lines U-1906-L and U-2020 were obtained from American Type Culture Collection (ATCC) and cultivated in RPMI-1640 Medium (Sigma-Aldrich Sweden AB, Stockholm, Sweden) supplemented with 10% FBS (Sigma-Aldrich) and l-Glutamine (2 mM, Sigma-Aldrich) and cells were maintained in 37 °C humidified air with 5% CO_2_. The cell lines were tested for mycoplasma during the project with MycoAlert™ Mycoplasma Detection Kit (Lonza).

### Cytotoxicity assay

The Fluorometric Microculture Cytotoxicity Assay (FMCA) was used as previously described [17] to investigate the in vitro effect of the PIs bortezomib and b-AP15 and the chemotherapeutic drugs with different mechanisms of action; the antimetabolite gemcitabine, the alkylating agent cisplatin, the antimitotic drug vinorelbine and the EGFR tyrosine kinase inhibitor gefitinib (all from Swedish Pharmacy). A successful FMCA assay required a ratio > 10 between the signal in the control and blank wells and a coefficient of variation < 30% in the control wells. Drug effect was defined as 1 minus the ratio of the number of live cells in a drug-treated sample compared with the number of cells in an untreated sample. IC_50_ values were determined from nonlinear regression of the dose–response relationships, with the GraphPad Prism software (GraphPad Software, Inc., CA, USA).

Combinations of the chemotherapeutic drugs and the PIs were evaluated by applying the median effect method, by Chou and Talalay [18], to the FMCA. Each combination was tested in two cell lines; the most sensitive and the most resistant to the therapeutic drug according to IC_50_-values determined from single drug experiments with the FMCA (Tables 1, 2). For gefitinib the second most resistant cell line was used due to that the IC_50_ value could not be determined. Drug concentrations used in the median effect method analysis were 0.25 ×, 0.5 ×, 1 ×, 2 ×, and 4 × the IC_50_ concentration for each drug. Very low (< 2.5%) and high (> 97.5%) SI-values were excluded from the calculations as recommended by Chou [19]. Combination indices (CIs) were calculated with the CalcuSyn software, version 2 (Biosoft, Cambridge, UK). The combination effects were graded with regard to their CIs according to the CalcuSyn manual.

**Table 1:** IC_50_ values [μM] for the drugs included in the cytotoxicity analyses in the LC cell line panel

	NCI-H23	NCI-H157	U-1752	U-1810	U-1906-L	U-2020
b-AP15	0.41	0.52	1.6	0.32	0.50	2.8
Bortezomib	0.018	0.012	0.057	0.088	0.083	0.036
Cisplatin	3.8	26	7.3	6.3	5.5	34
Gefitinib	270	130	> 310*	73	181	54
Gemcitabine	0.29	120	155	2.2	0.35	0.60
Vinorelbine	0.0032	0.041	4.2	0.007	0.0054	5.4

* Not determinable due to that the IC_50_ value was far above the tested concentration interval

**Table 2:** The CIs at 90% effect level of the drug combinations

Therapeutic drug	Inhibitor	Cell line	Cell line sensitivity to therapeutic drug	CI at 90% effect level	Effect
Cisplatin	Bortezomib	NCI-H23	Sensitive	1.30	Moderate antagonism
		U-2020	Resistant	0.61	Synergism
	b-AP15	NCI-H23	Sensitive	0.41	Synergism
		U-2020	Resistant	0.72	Moderate synergism
Gefitinib	Bortezomib	U-2020	Sensitive	ND	ND
		NCI-H23	Resistant	0.37	Synergism
	b-AP15	U-2020	Sensitive	ND	ND
		NCI-H23	Resistant	ND	ND
Gemcitabine	Bortezomib	NCI-H23	Sensitive	0.32	Synergism
		U-1752	Resistant	0.28	Strong synergism
	b-AP15	NCI-H23	Sensitive	ND	ND
		U-1752	Resistant	0.56	Synergism
Vinorelbine	Bortezomib	NCI-H23	Sensitive	0.94	Nearly additive
		U-2020	Resistant	0.38	Synergism
	b-AP15	NCI-H23	Sensitive	0.63	Synergism
		U-2020	Resistant	0.55	Synergism

*ND* not determinable due to poor curve fit by the CalcuSyn software

All drugs and drug combinations were tested in duplicates and each FMCA experiment was repeated at least two times.

## Results

### Drug combination analysis

The FMCA method was used to investigate the single agent activity of the chemotherapeutic drugs cisplatin, gefitinib, gemcitabine and vinorelbine and the PIs bortezomib and b-AP15 in six LC cell lines. The obtained IC_50_-values varied between the drugs and the cell lines, and are presented in Table 1 (the survival indices (SIs) used to calculate IC_50_ values are presented in Additional file 1).

**Fig. 1:**
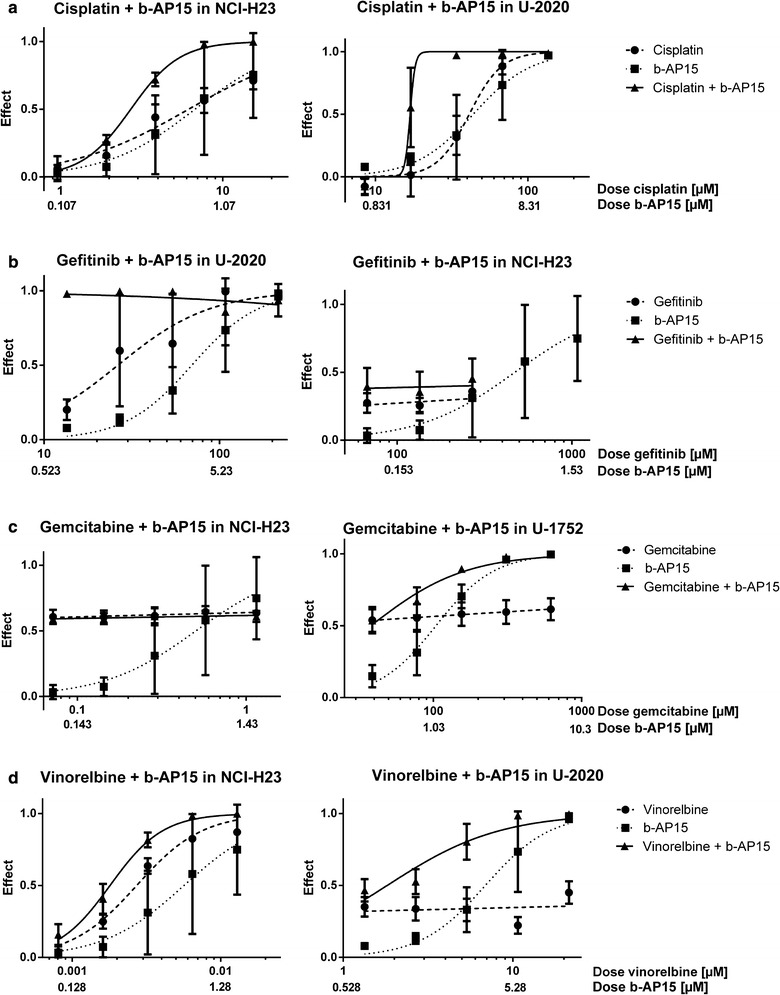
Dose response curves for cisplatin **a**, gefitinib **b**, gemcitabine **c** and vinorelbine **d** combined with b-AP15 in the least (left panel) and most (right panel) resistant cell line, in the cell line panel used in this study, in regard to the therapeutic drug. The effect is defined as 1 minus the fraction of living cells in a drug-treated sample compared with an untreated sample

**Fig. 2:**
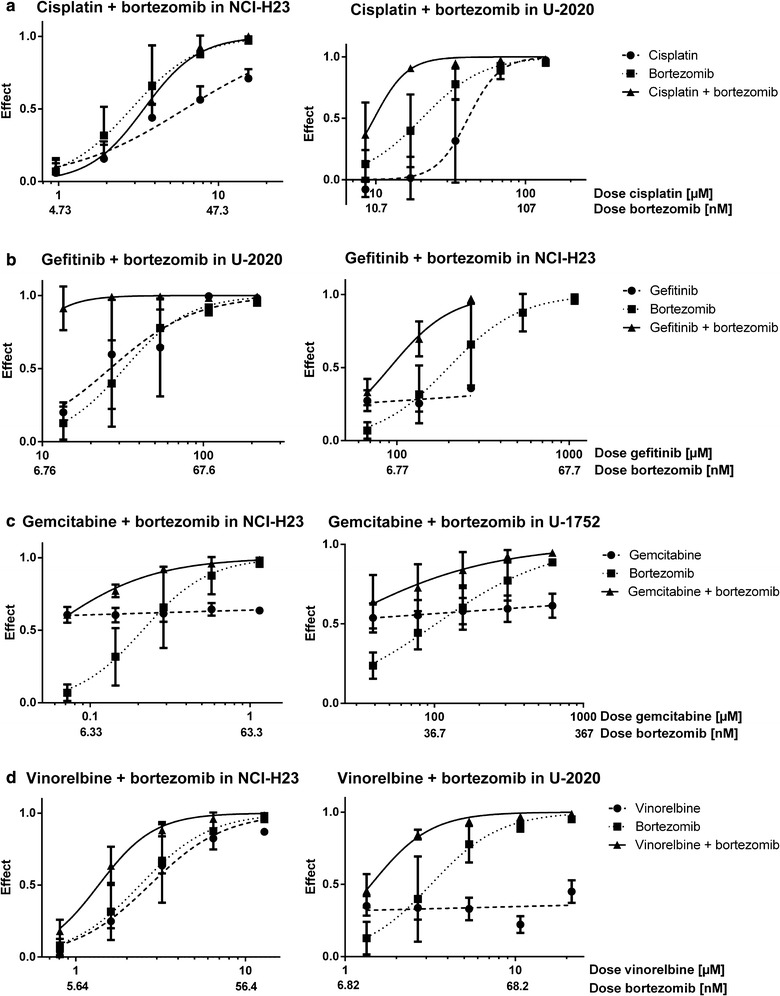
Dose response curves for cisplatin **a**, gefitinib **b**, gemcitabine **c** and vinorelbine **d** combined with bortezomib in the least (left panel) and most (right panel) resistant cell line, in the cell line panel used in this study, in regard to the therapeutic drug. The effect is defined as 1 minus the fraction of living cells in a drug-treated sample compared with an untreated sample

Two cell lines were selected for each therapeutic drug to be used in the drug combination analyses; the most resistant (highest IC_50_) and the most sensitive (lowest IC_50_-value) cell line in the panel (Tables 1, 2, the effect levels used to calculate the CIs are presented in Additional file 2). However, for the combinations with gefitinib the second most resistant cell line was used because the IC_50_ value was not possible to determine in the most resistant cell line since gefitinib had no effect on this cell line at the highest soluble concentration (310 µM). The outcomes of combining the PIs bortezomib and b-AP15 with each of the four chemotherapeutic drugs were evaluated with the median effect method [18]. The combinations with cisplatin, gemcitabine and vinorelbine had synergistic effects at the effect level of 90% in at least one of the tested cell lines (Table 2 and dose–response curves in Figs. 1, 2, the effect levels). The effects of gefitinib combined with b-AP15 could not be determined in any of the tested cell lines and gefitinib combined with bortezomib could not be determined in one of the cell lines, due to poor curve fit by the CalcuSyn software. Although all cell lines were very resistant to gefitinib (all IC_50_ values > 50 µM) the combination with either b-AP15 or bortezomib had close to 100% effect in the least gefitinib-resistant cell line U-2020 (Figs. 1b, 2b and Additional file 2), indicating that this is a very effective combination.

The effects of the two different PIs were similar in the tested cell lines. Bortezomib showed slightly higher combination effects than b-AP15 in the resistant cell lines, in regard to the therapeutic drugs, and slightly lower effects compared to b-AP15 in the sensitive cell lines, in regard to the therapeutic drugs (Figs. 1, 2, Table 2; Additional file 2). The combination of bortezomib with cisplatin, gemcitabine or vinorelbine showed more effects (lower CIs) in the resistant cell lines than the sensitive cell lines, in regard to the therapeutic drugs. This could not be seen for b-AP15. Since proteasomal inhibition causes proteotoxic stress with an increase in free radicals which increases the effect of DNA damaging agents, especially in resistant cells, this indicates that b-AP15 may induce lower levels of free radicals than bortezomib. Another difference between the inhibitors was that bortezomib combined with cisplatin had moderate antagonism in the cisplatin-sensitive cell line, whereas b-AP15 had synergistic effects in this cell line. The overall similar effects of the inhibitors indicate that the combination effects are due to specific proteasomal inhibition and not due to off-target effects.

## Discussion

LC is the leading cause of cancer deaths in the world and the prognosis for the patients is very poor. Hence, better treatment options are urgently needed for these patients. Combination chemotherapy has shown clinical benefit for various cancers [20], including LC [3]. PIs with their broad anti-tumor mechanism have shown promise in cancer therapy and after the introduction of bortezomib, the first PI in clinical use for multiple myeloma, second generation PIs have been developed as well as other inhibitors of the ubiquitin–proteasome system [21]. In this study we did a drug combination screening by combining chemotherapeutic drugs used in the clinic for LC patients, representing different mechanisms of action, with two different PIs. We found that cisplatin, gemcitabine and vinorelbine had synergistic effects when combined with either of the PIs and that the PIs potentiated the effect of gefitinib in LC cells.

All cell lines were highly resistant to gefitinib in vitro (all IC_50_ values > 50 µM). Resistance to gefitinib is strongly connected with the mutation status of EGFR [22]. To our knowledge the mutational status of EGFR is only known in three of the cell lines in our study; NCI-H23, U-1752 and NCI-H157, which all have wild-type EGFR [23–25]. NCI-H157 was one of the least resistant cell lines to gefitinib (IC_50_ value below median), whereas NCI-H23 was the second most resistant cell line and U-1752 the most resistant cell line (IC_50_ > 310 µM) to gefitinib, indicating that other factors than EGFR mutation status may also influence the sensitivity to gefitinib. Anyhow, gefitinib combined with PIs had close to 100% effect even at the lowest tested concentrations (0.25 × IC_50_ for each drug) in the least resistant cell line U-2020. Due to the high effect at all tested concentrations for gefitinib the level of synergy could not be determined with the Calcusyn Software [19]. Hence, the combinations are highly potent in the gefitinib-resistant cell lines used in our study, and warrants further analysis to establish their potential as new treatment combinations for lung cancer. With studies analyzing the mechanism of the potent effect in these cell lines the combination could potentially be used to develop individualized therapy for lung cancer patients.

Comparing the combination effects of the PIs showed that they had similar effects. However, bortezomib combined with cisplatin, gemcitabine and vinorelbine had more effects in the resistant cells compared with the more sensitive cells, in regard to each therapeutic drug. This could not be seen for b-AP15. Since proteasomal inhibition causes proteotoxic stress with an increase in free radicals which increases the effect of DNA damaging agents, especially in resistant cells, this indicates that b-AP15 may induce lower levels of free radicals than bortezomib and that bortezomib may be a more effective second-line treatment than b-AP15 for LC patients who have become resistant to DNA damaging agents.

There are several preclinical and clinical studies indicating that the drug combinations tested in our study have potential for the treatment of LC patients. Bortezomib potentiates the effect of cisplatin in different cancer cell types [26, 27] and induces apoptosis in cisplatin-resistant SCLC cells [28]. The combination of bortezomib with radiation therapy and cisplatin was shown in a phase I trial to be safe for treatment of head and neck cancer patients [29]. A phase I/II trial of bortezomib in combination with the alkylating agent bendamustine has shown promising efficacy in relapse multiple myeloma patients [30]. The combined treatment of bortezomib and EGFR inhibitors has a synergistic growth inhibitory and pro-apoptotic activity in different human cancer cells [31] and bortezomib inhibits the growth of gefitinib-resistant NSCLC cells [32]. In a phase II trial of relapsed NSCLC patients, the combination of bortezomib and the EGFR inhibitor erlotinib did not show any survival benefit compared with erlotinib alone [33]. However, it is possible that due to the small size of this study (n = 57) any benefits of adding bortezomib to the treatment with erlotinib could not be detected. Due to the high efficacy levels of the combination of bortezomib and EGFR inhibition in the LC cell lines in our study we believe that further studies of this combination are warranted in order to establish its potential as a new treatment option for lung cancer. Bortezomib potentiates the effect of gemcitabine in NSCLC cells [34], and bortezomib combined with gemcitabine has shown promise in clinical trials for patients with refractory peripheral T cell lymphoma [35] and refractory mantle cell lymphoma [36]. To our knowledge, no clinical study with single agent vinorelbine in combination with a PI has been done, but a phase II trial of bortezomib combined with docetaxel showed no survival benefit compared with bortezomib alone in patients with advanced NSCLC [37]. Since the mechanisms of action for vinorelbine and docetaxel differs, vinorelbine inhibits microtubules [38] whereas docetaxel promotes and stabilizes the microtubule assembly [39], it is possible that the combination of bortezomib with an antimitotic drug which inhibits microtubule assembly has better survival benefits for LC patients.

Since drug resistance is a major clinical problem in LC, as well as in other tumor types, there may be a rationale to concomitantly combine treatment with PIs with other chemotherapeutic agents upfront in order to prevent or delay resistance from occurring. Our study also demonstrates the synergistic action when combining the drugs concomitantly and would argue against using PIs and chemotherapeutics in sequence. This concomitant approach is also supported by results from other studies in vitro and in vivo. Although, further studies evaluating the potential of increasing the synergistic effects by sequential administration would be interesting.

It is possible that combinations of more than two of the agents that were used in this study may have even stronger anti-tumor effects than combinations with two agents. For example, a Children’s Oncology Group clinical trial investigated if bortezomib increased the efficacy of ifosfamide and vinorelbine in paediatric Hodgkin lymphoma and demonstrated a promising response rate with the triple combination [40]. Bortezomib combined with gemcitabine and carboplatin has demonstrated survival benefits for NSCLC patients [41]. However, in another phase II trial in locally advanced or metastatic NSCLC the addition of bortezomib to standard chemotherapy with cisplatin and gemcitabine did not demonstrate an additional clinical benefit [42]. Hence, further studies are needed to evaluate the effect of drug combinations with three or more drugs in LC.

## Conclusions

PIs combined with chemotherapeutic agents used in routine treatment for LC patients have synergistic effects in LC cells in vitro. Clinical trials have indicated that these combinations are safe for cancer patients and may provide benefit for LC patients. However, further in vitro and in vivo studies of these combinations are needed to establish the optimal combination strategies and to confirm its efficacy and safety in larger clinical trials with LC patients.

## Additional files

**Additional file 1.** SIs for each replicate of the FMCA experiments of single drugs. SI is defined as the fraction of living cells in a drug treated sample compared with an untreated sample. Each replicate is a mean of the duplicates in each experiment.

**Additional file 2.** Effect levels for each replicate of the FMCA experiments of the drug combinations. Effect is defined as 1 minus the fraction of living cells in a drug treated sample compared with an untreated sample. Each replicate is a mean of the duplicates in each experiment.

## Abbreviations

CIs: combination indices
EGFR: epidermal growth factor receptor
FMCA: Fluorometric Microculture Cytotoxicity Assay
LC: lung cancer
NSCLC: non-small cell lung cancer
PI(s): proteasome inhibitor(s)
SCLC: small cell lung cancer
SI(s): survival index (indices)
